# Chronic Adolescent Restraint Stress Downregulates miRNA-200a Expression in Male and Female C57BL/6J and BALB/cJ Mice

**DOI:** 10.3390/genes15070873

**Published:** 2024-07-03

**Authors:** Helen M. Kamens, Emma K. Anziano, William J. Horton, Sonia A. Cavigelli

**Affiliations:** Department of Biobehavioral Health, The Pennsylvania State University, University Park, PA 16801, USAwjh23@psu.edu (W.J.H.); sac34@psu.edu (S.A.C.)

**Keywords:** adolescent stress, miR-200a, microRNA-200a, microRNA

## Abstract

Adolescence is a critical developmental period when the brain is plastic, and stress exposure can have lasting physiological consequences. One mechanism through which adolescent stress may have lasting effects is by altering microRNAs (miRNAs), leading to wide-scale gene expression changes. Three prior independent studies used unbiased approaches (RNA sequencing or microarray) to identify miRNAs differentially expressed by chronic variable stress in male rodents. In all three studies, miRNA-200a was differentially expressed in areas of the brain associated with emotion regulation. The current study extends this research to determine if chronic non-variable adolescent stress downregulates miRNA-200a expression by looking at two strains (BALB/cJ and C57BL/6J) of male and female mice. We utilized a 14-day (2 h/day) restraint stress protocol and verified stress effects on adolescent body weight gain and circulating corticosterone concentrations relative to non-restraint controls. Mice were then left undisturbed until they were euthanized in adulthood, at which time brains were collected to measure miRNA-200a in the ventral hippocampus. Three weeks after adolescent stress ended, differences in body weight between groups were no longer significant; however, animals exposed to stress had less miRNA-200a expression in the ventral hippocampus than control animals. These data implicate miRNA-200a expression as a potential mechanism by which adolescent stress can have persistent impacts on multiple outcomes in both male and female mice.

## 1. Introduction

Adolescence is a key developmental period when environmental exposures can have long-term consequences. Stress is impactful during this time because the brain is structurally plastic as it develops and matures. Research suggests that adolescent environmental exposures, including stress, may result in epigenetic reprogramming [1]. Such reprogramming can alter structural and functional properties of the brain.

One epigenetic mechanism that has received increased attention is a class of noncoding RNAs called microRNAs (miRNAs). miRNAs play a role in synaptic plasticity, making them a compelling target for the lasting effects of stress exposure [2]. Several papers have examined miRNA responses to stress [3,4]; however, most of these studies examined targeted miRNA based on biological pathways linked to stress physiology (e.g., those related to hypothalamic–pituitary–adrenal (HPA) function). Fewer studies have taken an unbiased approach and examined global changes in miRNA through microarray or RNA sequencing. While there are fewer unbiased studies, they provide the advantage of being able to identify novel genes and biological pathways altered by stress.

Based on three unbiased studies examining effects on miRNA expression, miR-200a has been identified as altered by chronic stress. miRNA-200a-3p and miRNA-200a-5p are derived from the miRNA-200a hairpin precursor [5]. Our lab observed that miRNA-200a-3p and miRNA-200a-5p were downregulated in the prefrontal cortex of adult (postnatal day (PND) 70) male C57BL/6J mice exposed to 5 weeks of chronic variable social stress during adolescence (PND 25–59) [6]. In the next study, male Sprague-Dawley rats exposed to chronic unpredictable mild stress in adulthood (>56 PND) revealed time-dependent effects in the prefrontal cortex; miR-200a-3p was downregulated after 4 weeks of stress, but when stress lasted for 12 weeks, this gene was upregulated [7]. Finally, Liu and colleagues used a microarray to examine miRNA regulation in the hippocampus of male ICR mice exposed to chronic unpredictable mild stress for 4 weeks in adolescence (PND 35–63). Here, miR-200a-3p was upregulated in mice exposed to stress versus control conditions [8]. A key difference in the Liu study was that following chronic unpredictable mild stress, but before tissue was taken for gene expression analyses, both stress and control animals were isolate housed and exposed to 24-h water/food deprivation for sucrose preference testing. Therefore, control animals were also exposed to known rodent stressors (i.e., isolation, food/water deprivation) prior to tissue dissection for miRNA expression measurement. Given that this miRNA was identified in three independent, unbiased studies, these data support the role of miRNA-200a as a possible mediator of multiple changes following stress.

In the current study, we extended these findings by taking a targeted approach and examining the expression of miRNA-200a after non-variable chronic adolescent restraint stress. We used a chronic restraint protocol because it has been shown to affect adolescent male BALB/cAnNCrl mice [9]. Further, the adolescent HPA axis does not completely habituate to this repeated homotypic stressor [9,10] and it can cause long-lasting peripheral effects (e.g., cardiac function) that are not observed with the commonly used chronic variable stress procedures [11]. The goal of this research was to determine if this non-variable stressor during adolescence can also lead to persistent downregulation of this miRNA in an area of the brain that regulates multiple emotion and stress regulatory responses including the HPA axis (ventral hippocampus [12]). We hypothesized that 2 weeks of chronic adolescent restraint stress would decrease the expression of miRNA-200a in the ventral hippocampus. Given the prior differences in findings with this gene, we chose to examine the sole effects of chronic stress. Thus, the animals were not exposed to other manipulations between adolescent restrain stress and collection of tissue for miRNA expression. We further extended prior research by examining the effects of stress on miRNA-200a gene expression in female animals and in two inbred strains of mice that have been shown to respond differently to chronic stress [6,13].

## 2. Materials and Methods

### 2.1. Animals

Adolescent (PND 28) male and female C57BL/6J and BALB/cJ mice were obtained from The Jackson Laboratory. Mice were group housed with mice of the same sex and strain and remained undisturbed for 9 days. Animals were housed (3/cage) in standard polycarbonate cages with corncob bedding and a shredded paper nestlet. The animals were housed in a temperature and humidity controlled room on a 12-h light–dark schedule (lights on at 0700 h). Ad libitum food and water were available. The Pennsylvania State University IACUC committee approved all procedures (protocol #PROTO202102117).

### 2.2. Restraint Stress and Body Weight

Cages were randomly assigned to either restraint stress or control conditions. The stress animals underwent 14 consecutive days of restraint stress (PND 37–50) [9]; each day, stress animals were moved from the colony room into a testing room, removed from their home cage, weighed, and placed in a 50 mL conical tube perforated with air holes for 2 h. Mice were restrained daily from 900–1100 h. After each restraint session, the stress mice were returned to their home cage in the colony room. Measures were taken to minimize all other sources of variability between stress and control mice, including control mice being handled daily by the investigators to obtain weights in the colony room during each of the 14 days when the experimental animals experienced stress. Additionally, the experimenters changed both control and stress cages on the same schedule. Finally, all mice were weighed again by the investigators on the day they were euthanized.

### 2.3. Blood Collection and Corticosterone Assay

On the 14th day of stress (PND 50), blood was taken to measure circulating glucocorticoid levels at the end of the 2-h restraint period. The tip of the tail was cut, and blood samples were collected into heparinized capillary tubes (Ram Scientific, Austell, GA, USA) and then stored on ice. Blood from control mice was taken at the same time as from the stress animals (~1100–1200 h). For control mice, individual cages were brought into the testing room to collect the sample within 3 min of disruption. The blood was centrifuged for 10 min at 3000× *g* at 4 °C. Plasma was stored at −20 °C until analysis with a commercial corticosterone ELISA assay (Arbor Assay, Ann Arbor, MI, USA) following the manufacturer’s protocol. Intra- and inter-assay coefficients of variation were 2.7–5.8 and 4.6, respectively.

### 2.4. Brain Extraction, RNA Isolation, and qPCR

Following the restraint stress protocol, the mice remained undisturbed for an average of 24 days until they were euthanized (between PND 69 and 77). This time was chosen as it is consistent with the ages at which effects of adolescent stress are seen on behavioral outcomes in male and female C57BL/6J and BALB/cJ mice [14,15,16] and was similar to when we observed changes in miRNA200a following chronic variable social stress in male C57BL/6J mice [6]. Mice were euthanized by cervical dislocation, brains were removed and sliced in a brain matrix, and the ventral hippocampus was taken with a Harris Uni-CoreTM punch. The ventral hippocampus from both hemispheres was placed into RNAlater (Ambion, Austin, TX, USA). RNA was extracted using the Qiagen RNeasy^®^ Plus Universal Mini Kit according to the manufacturer’s guidelines for extracting total RNA, including small RNAs. Total RNA served as the template for reverse transcription to complementary DNA (cDNA) using the High-Capacity cDNA Archive Kit (Applied Biosystems, San Francisco, CA, USA). Expression of miRNA-200a and β-actin (*Actb*; control gene) were analyzed with validated TaqMan expression assays (Applied Biosystems).

Quantitative PCR reactions were run in a QuantStudio 3 Real-Time PCR system. All samples were run in triplicate, and the average cycle threshold (Ct) for each gene was used to calculate relative expression based on the 2^−ΔΔCt^ method presented as fold change relative to male control C57BL/6J mice.

### 2.5. Bioinformatics Analysis

Predicted mRNA targets of miRNA-200a were obtained from DIANA-microT 2023 at an interaction threshold of ≥0.7 [17]. This list of genes was then analyzed in QIAGEN Ingenuity Pathway Analysis (IPA) to identify enriched canonical pathways [18]. Full IPA analysis settings are available in Table S1.

### 2.6. Statistics

Data were analyzed in SPSS with factorial ANOVA. Where appropriate, independent factors included condition (control, restraint), strain (BALB/cJ, C57BL/6J), sex (male, female), and day of stress (1–14). Age at sacrifice was included as a covariate in the gene expression and body weight at sacrifice ANOVAs. ANOVAs with fewer factors were conducted to decompose interactions with multiple factors. Significant 2-way interactions were interpreted with simple effects post hoc with Bonferroni correction. The α was set to 0.05. Data are reported as mean ± SEM. Full data in this manuscript can be found in Table S6.

## 3. Results

### 3.1. Body Weight

#### 3.1.1. Day 1

Analysis of body weight from day 1, before stress exposure, indicated no significant baseline differences in body weight between stress and control animals (Figure S1). Male mice weighed more than female mice (main effect of sex (F_1, 39_ = 113.4, *p* < 0.001); 21.8 g ± 0.2, 18.1 g ± 0.2, respectively), but there were no significant strain, condition, or interaction effects.

#### 3.1.2. Days 2–14

**Figure 1:**
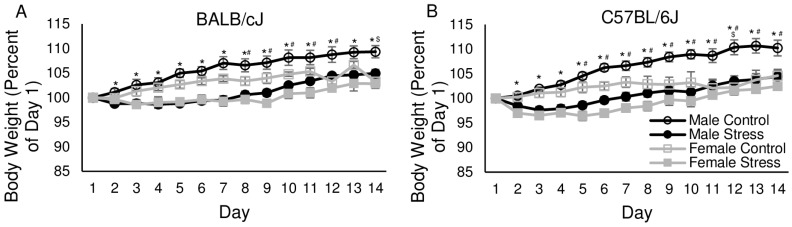
Adolescent body weight depicted as a percentage of day 1 weight across 14 days of restraint stress in male and female BALB/cJ (**A**) and C57BL/6J (**B**) mice. *N* = 5–6/group. * = *p* < 0.05 main effect of condition, # = *p* < 0.05 main effect of sex, $ = *p* < 0.05 condition X sex interaction.

In both BALB/cJ and C57BL/6J mice, exposure to chronic restraint in adolescence led to slower weight gain than control conditions. To examine the effect of stress on body weight, the percentage of day 1 weight was calculated for each of the 14 days of restraint (Figure 1); thus, values greater than 100 represent weight gain, while values less than 100 indicate weight loss relative to the first day of the experiment. A repeated measures analysis including strain, sex, condition, and day as factors revealed several main effects and interactions, including a strain X sex X condition X day interaction (F_13, 507_ = 1.9, *p* < 0.05); thus, we further analyzed each strain independently.

In BALB/cJ mice, an ANOVA including day, sex, and condition as factors identified a significant main effect of day (F_13, 247_ = 53.4, *p* < 0.001), day X sex (F_13, 247_ = 5.9, *p* < 0.001), and day X condition (F_13, 247_ = 9.7, *p* < 0.001) interactions. Thus, as follow-up analyses, an ANOVA was performed on data from each day, using the percentage body weight change from day 1 as the dependent variable. Beginning on the second stress day, BALB/cJ mice exposed to restraint stress gained less weight than control animals (Figure 1; main effect of condition on all days (*p* < 0.05); see full ANOVA results in Table S2). On days 8–12, male BALB/cJ mice gained weight more rapidly than female BALB/cJ mice (Figure 1; main effect of sex on days 8–12 (*p* < 0.05)). On day 14, stressed male BALB/cJ gained significantly less weight than control male BALB/cJ mice (significant sex X condition interaction (F_1, 19_ = 4.5, *p* < 0.05); post hoc *p* < 0.01). There were no significant differences between female stress and control BALB/cJ animals on day 14.

In C57BL/6J animals, adolescent restraint stress similarly affected body weight. Similar to BALB/cJ mice, in the omnibus analysis, there were several main effects and interactions (main effect of day (F_13, 260_ = 37.4, *p* < 0.001), day X sex (F_13, 260_ = 4.9, *p* < 0.001), day X condition (F_13, 260_ = 5.2, *p* < 0.001), day X sex X condition interactions (F_13, 260_ = 2.7, *p* < 0.01)); thus, follow-up ANOVAs were conducted with data from each day. From the second day, C57BL/6J mice exposed to restraint stress gained less weight than control mice (Figure 1; main effect of stress (*p* < 0.05); see full ANOVA results in Table S3). Also similar to BALB/cJ mice, C57BL/cJ female mice gained weight more slowly than male C57BL/6J mice (main effect of sex on days 5–14 (*p* < 0.05)). Finally, on day 12, stressed male C57BL/6J mice gained significantly less weight than control male C57BL/6J mice (significant sex X condition interaction (F_1, 20_ = 6.1, *p* < 0.05); post hoc *p* < 0.01), a difference that was not observed in female C57BL/6J animals.

#### 3.1.3. In Adulthood at Sacrifice

**Figure 2:**
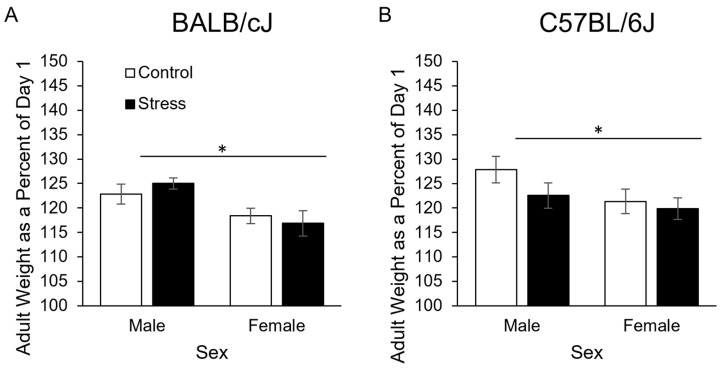
Adult body weight depicted as a percentage of day 1 weight in male and female BALB/cJ (**A**) and C57BL/6J mice (**B**). *N* = 5–6/group. * = *p* < 0.05 main effect of sex.

Three weeks after stress ended (in adulthood), there were no longer differences in body weight based on experimental conditions (Figure 2). A three-way ANOVA indicated that male mice had gained significantly more weight (percentage of day 1 weight) than female mice (main effect of sex (F_1, 38_ = 14.1, *p* < 0.001);124.6 ± 1.1, 119.1 ± 1.1, respectively). However, no other significant effects or interactions were detected.

### 3.2. Corticosterone

**Figure 3:**
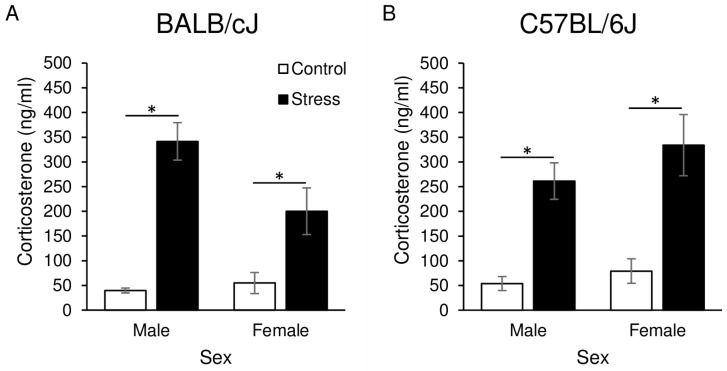
Circulating corticosterone (ng/mL) concentrations immediately after the conclusion of the day 14 restraint session in adolescent male and female BALB/cJ (**A**) and C57BL/6J (**B**) mice. *N* = 5–6/group. * = *p* < 0.05 main effect of condition.

Immediately after the final restraint stress exposure, corticosterone was significantly elevated in mice exposed to stress relative to control animals (Figure 3). A three-way ANOVA with strain, sex, and condition revealed a significant main effect of condition (F_1, 39_ = 77.5, *p* < 0.001) and a strain X sex interaction (F_1, 39_ = 4.7, *p* < 0.05). Mice exposed to restraint stress had elevated corticosterone compared to control mice, an effect that was independent of both strain and sex. There were, however, subtle differences between the strains. There was a statistical trend (post hoc *p* = 0.1) toward male BALB/cJ mice having higher corticosterone levels compared to female BALB/cJ mice (independent of stress condition, 204.3 ng/mL ± 170.9, 127.6 ng/mL ± 114.3, respectively). In contrast, male and female C57BL/6J mice had similar corticosterone levels (157.6 ng/mL ± 36.5, 206.6 ng/mL ± 49.9, respectively).

### 3.3. Gene Expression

**Figure 4:**
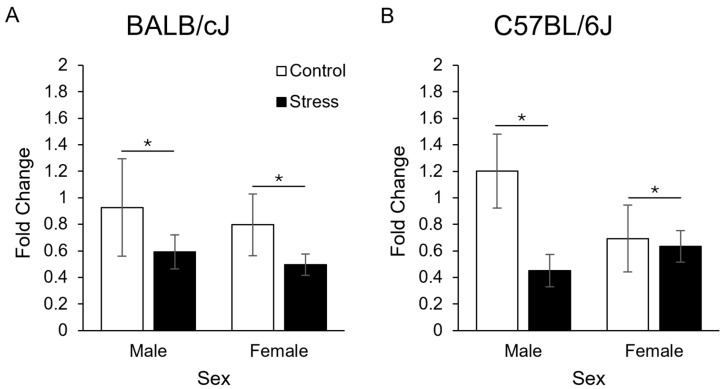
Fold change expression of miRNA-200a in adult male and female BALB/cJ (**A**) and C57BL/6J (**B**) mice. Data are combined on sex as there were no main effects or interactions with this variable. *N* = 5–6/group. * = *p* < 0.05 main effect of condition.

Three weeks after adolescent restraint stress had ended, there was decreased ventral hippocampal miRNA-200a expression in adult mice exposed to chronic restraint stress in adolescence (Figure 4; main effect of condition (F_1, 38_ = 7.3, *p* < 0.05)). No other significant main or interaction effects were observed (all *p* ≥ 0.17).

### 3.4. Bioinformatics Analysis

miRNA regulate the expression of many mRNAs. Using DIANA-microT 2023, we identified 1345 mRNAs that are predicted to be targeted by miRNA-200a at an interaction score of ≥ 0.7 (Table S4). To determine if these genes were enriched in specific biological pathways, IPA was used. Table 1 lists the top 10 canonical pathways identified by IPA (full output in Table S5). The top pathway identified was the pyridoxal 5′-phosphate salvage pathway. Pyridoxal 5′-phosphate is an active form of vitamin B6; it acts as a cofactor for more than 100 enzymatic reactions, including in the synthesis of several neurotransmitters [19]. For example, it acts as a cofactor for dopa decarboxylase, which is required for synthesizing dopamine and serotonin [20]. Another top pathway identified was RHOA signaling. Ras homolog gene family member A (RhoA) is a GTPase involved in several cellular functions with a high level of expression in the brain [21]. While these represent two specific pathways, other identified general pathways could be important in the lasting response to adolescent stress, including DNA methylation and transcriptional repression signaling, synaptogenesis signaling pathway, and gap junction signaling.

## 4. Discussion

The present study examined the effect of adolescent restraint stress on the expression of miRNA-200a in male and female BALB/cJ and C57BL/6J mice. To verify that 14 days of non-variable restraint led to continued stress and activation of the HPA axis in adolescent male and female BALB/cJ and C57BL/6J mice, we measured body weight and circulating corticosterone. We found that mice exposed to adolescent restraint stress gained less weight than control animals after a single 2-h restraint session; however, these body weight differences were no longer present 3 weeks after restraint ended. Although body weight had recovered 3 weeks after the conclusion of stress, expression of miRNA-200a in the ventral hippocampus was downregulated in stress-exposed mice at this time. These data indicate that miRNA-200a is downregulated 3 weeks after animals were exposed to a non-variable stressor in adolescence. Bioinformatic analysis suggests that mRNAs targeted by miRNA-200a regulates pathways involved in neurotransmitter synthesis and other critical brain processes.

miRNAs have received increased attention as a potential biological process by which stress exposure can have lasting effects. miRNA-200a and its two mature sequences, miRNA-200a-3p and miRNA-200a-5p, have been identified in prior studies from three independent labs that used unbiased approaches to examine miRNA expression after chronic stress. However, to date, this altered expression of miRNA-200a has only been observed in male rodents following 4+ weeks of variable stress. Here, we provide evidence that just 2 weeks of non-variable stress during adolescence decreases miRNA-200a expression in both male and female BALB/cJ and C57BL/6J mice. These data agree with findings from male C57BL/6J mice [6] and male Sprague-Dawley rats [7]. However, our data do not match results from existing literature on male ICR mice that showed that chronic, unpredictable mild stress during adolescence increased miRNA-200a-3p expression [8]. Several factors differ between our work and that of Liu and colleagues. Our research specifically examined the effect of only restraint stress on gene expression. In the work by Liu, following the experimental stress manipulation, all mice (stress and control animals) were isolate housed for sucrose preference testing that included a 24-h food and water deprivation period. Both social isolation and food/water deprivation are stressors in rodents [22,23]; thus, even control animals experienced stress. Notably, an acute (2-h) stressor is known to change miRNA expression [24]. Therefore, it is possible that the isolation and food/water restriction stress in the Liu study may have altered miRNA-200a expression.

Inconsistent findings may also be a result of well-documented differences in stress sensitivity across strains of mice. For example, a study that directly compared outbred ICR mice to inbred C57BL/6 mice observed strain differences in depression phenotypes after chronic mild stress, with only C57BL/6 mice demonstrating stress-induced depressive-like behavior when measured by the forced swim test [25]. A goal of RNA sequencing and microarray research is to identify novel genes involved in traits of interest. The current data extend three such studies that have indicate miRNA-200a in stress responsivity. These data indicate that this gene is downregulated in response to chronic non-variable stress in C57BL/6J and BALB/cJ mice. While we cannot exclude the possibility that the physiological stress response may differ in other mouse strains, including ICR mice, it seems plausible that miRNA-200a is a broad mechanism by which stress induces lasting changes in brain functionality.

In our gene expression analysis, it is important to note that we only detected a statistically significant effect of stress condition. All main effects or interactions with sex were not significant (*p* ≥ 0.17). However, when examining the gene expression results presented split by strain and sex in Figure 4, we note that this effect may not be present in female C57BL/6J mice. Additional research should more fully examine this relationship.

Beyond rodents, some data suggest that miRNA-200a is influenced by stress in other species. In a study using juvenile European seabass (*Dicentrarchus labrax*), miRNA-200a-3p was shown to correlate with cortisol production and recovery in response to an acute (2-min) confinement challenge protocol [26]. In humans, we know of no specific studies that have identified miRNA-200a as responsive to stress; however, miRNA-200a belongs to a family of five evolutionarily conserved genes known as the miRNA-200 family (including miRNA-200a, -200b, -200c, -141, and -429) [27]. In a study of the effects of childhood trauma exposure, miRNA-200b-5p was found to be downregulated [28]. These findings parallel our earlier work in male C57BL/6J mice where this miRNA (miRNA-200b-3p) was downregulated by an adolescent social stressor in addition to miRNA-200a [6]. Thus, while the current data suggest a role of miRNA-200a, it is also possible that there is a more generalized role of the miRNA-200 family of genes in response to stress.

There are more than 1300 mRNAs that are predicted to interact with miRNA-200a (see Table S4). These mRNA targets of miRNA-200a are involved in several pathways that may mediate how the brain responds to stress. One interesting pathway identified was the RhoA signaling pathway. RhoA is a GTPase involved in the destabilization of dendrites and has been linked to stress-induced behavioral changes. In adult mice exposed to social defeat stress, RhoA was upregulated in dopamine 1 medium spiny neurons in the nucleus accumbens compared to control animals. Further, a RhoA inhibitor injected during social defeat blocked effects on depressive-like behaviors [29]. Similar work using a prenatal stress model showed increased RhoA expression in the hippocampus and prefrontal cortex of animals exposed to stress [30]. It is possible that changes in miRNA-200a may mediate some of these effects, and future studies should examine this hypothesis.

Here, we report that after 14 days of restraint stress, corticosterone was elevated in adolescent BALB/cJ and C57BL/6J mice immediately after the final 2-h restraint session. We report this as an important verification of the model. The restraint stress protocol we adopted was initially described by Sadler and Bailey, who used a 14-day protocol and demonstrated elevated corticosterone in adolescent male BALB/cAnNCrl mice [9]. This work extends these findings to a different BALB substrain (BALB/cJ), C57BL/6J mice, and females of both strains. In adolescent BALB/c and C57BL/6 mice, exposure to a single 30-min restraint stress results in elevated corticosterone [31]. While we tried to decrease potential sources of variability by having similar handling and husbandry between stress and control groups, it is possible that there were subtle variations between groups that contributed to the findings we attribute to stress in this paper. We know that even within the lab environment there are multiple factors that can influence behaviors [32]. However, we believe these data suggest that chronic daily restraint stress elicits a physiological stress response in adolescent mice that does not fully habituate even after 14 days of restraint. Moreover, this is true in both male and female BALB/cJ and C57BL/6J mice.

In a second verification of the two-week restraint stress model, male and female mice exposed to chronic stress had slower body weight gain relative to control animals. In our data, BALB/cJ and C57BL/6J mice exposed to restraint stress exhibited reduced body weight gain after a single restraint session (i.e., on day 2). These data are similar to other adolescent stress exposure findings. For example, male C57BL/6J mice weighed less after a single social defeat exposure than control animals [33]. However, our data in female mice does not agree with prior literature. No difference in body weight was observed in adolescent female C57BL/6J mice exposed to accelerated social defeat compared to control animals [34]. Moreover, in Wistar rats, mixed-modality psychosocial stress (social isolation, social defeat, and restraint) during adolescence resulted in sex-dependent differences in body weight. Male rats exposed to stress had decreased body weight gain, but this was not the case in female rats [35]. Multiple factors differ between our work and these prior findings, including stressor, species, and exposure age, that could contribute to these observed differences in results. While stress mice gained weight more slowly than control animals, 3 weeks after the stressor ended, there were no longer differences in body weight between stress and control conditions, suggesting that growth recovers following chronic adolescent stress but that downregulation of ventral hippocampus miRNA-200a expression persists.

This study aimed to build on existing literature indicating that miRNA-200a expression is altered by chronic stress. Here, we verified that 14 consecutive days of restraint is a robust stressor in adolescent male and female BALB/cJ and C57BL/6J mice. Moreover, we show decreased expression of miRNA-200a in mice exposed to stress approximately 3 weeks after stress ended. This is in contrast to the differences in body weight that recovered by this time. Adolescent stress is a known risk factor for many maladaptive responses; future work should determine if changes in miRNA-200a expression is a causal mechanism.

## Figures and Tables

**Table 1:** IPA canonical pathways.

IPA Top 10 Canonical Pathways	-log(*p*-Value)
Pyridoxal 5′-phosphate Salvage Pathway	6.86
Epithelial Adherens Junction Signaling	4.6
Salvage Pathways of Pyrimidine Ribonucleotides	4.53
DNA Methylation and Transcriptional Repression Signaling	4.4
Synaptogenesis Signaling Pathway	4.02
Pulmonary Healing Signaling Pathway	3.19
Gap Junction Signaling	3.19
GNRH Signaling	2.9
RHOA Signaling	2.78
HOTAIR Regulatory Pathway	2.76

Top 10 canonical pathways identified by IPA.

## Data Availability

The original contributions presented in the study are included in the article/Supplementary Materials, further inquiries can be directed to the corresponding author.

## Supplementary Materials

The following supporting information can be downloaded at: https://www.mdpi.com/article/10.3390/genes15070873/s1, Figure S1: Baseline body weight in male and female BALB/cJ and C57BL/6J mice; Table S1: Full IPA analysis settings; Table S2: Full ANOVA results in BALB/cJ mice; Table S3: Full ANOVA results in C57BL/6J mice; Table S4: List of mRNAs predicted to be targeted by miRNA-200a from DIANA-microT 2023; Table S5: All canonical pathways identified by QIAGEN Ingenuity Pathway Analysis (IPA); Table S6: Raw data included in this manuscript.
