# Supplementary material for: Chronic Adolescent Restraint Stress Downregulates miRNA-200a Expression in Male and Female C57BL/6J and BALB/cJ Mice
Source: Genes (Basel). 2024 Jul 3;15(7):873. doi: 10.3390/genes15070873 (PMC11275362; doi:10.3390/genes15070873)
Supplement: Supplementary file 1 [file genes-15-00873-s001.zip › miRNA200a Figure S1.pdf]

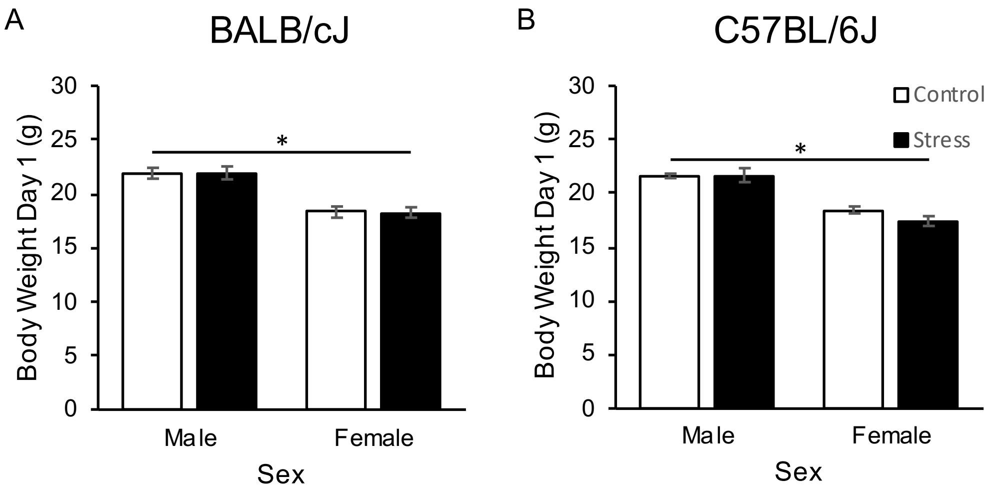

Supplementary Figure S1 Body weight on day 1 in male and female (A) BALB/cJ and (B) C57BL/6J mice. $N = 5\text{-}6$/group. * = $p < 0.05$ main effect of sex
