# Supplementary material for: Chronic Adolescent Restraint Stress Downregulates miRNA-200a Expression in Male and Female C57BL/6J and BALB/cJ Mice
Source: Genes (Basel). 2024 Jul 3;15(7):873. doi: 10.3390/genes15070873 (PMC11275362; doi:10.3390/genes15070873)
Supplement: Supplementary file 1 [file genes-15-00873-s001.zip › miRNA200a Table S2.pdf]

	Main Effect of Day	Main Effect of Sex	Day X Sex Interaction
Day 2	$F_{1, 19} = 5.2$, $P < 0.05$	NS	NS
Day 3	$F_{1, 19} = 14.9$, $P < 0.01$	NS	NS
Day 4	$F_{1, 19} = 16.7$, $P < 0.001$	NS	NS
Day 5	$F_{1, 19} = 49.2$, $P < 0.001$	NS	NS
Day 6	$F_{1, 19} = 40.7$, $P < 0.001$	NS	NS
Day 7	$F_{1, 19} = 42.1$, $P < 0.001$	NS	NS
Day 8	$F_{1, 19} = 27.7$, $P < 0.001$	$F_{1, 19} = 5.0$, $P < 0.05$	NS
Day 9	$F_{1, 19} = 33.2$, $P < 0.001$	$F_{1, 19} = 7.3$, $P < 0.05$	NS
Day 10	$F_{1, 19} = 27.8$, $P < 0.001$	$F_{1, 19} = 7.6$, $P < 0.05$	NS
Day 11	$F_{1, 19} = 24.2$, $P < 0.001$	$F_{1, 19} = 7.9$, $P < 0.05$	NS
Day 12	$F_{1, 19} = 7.0$, $P < 0.05$	$F_{1, 19} = 14.1$, $P < 0.01$	NS
Day 13	$F_{1, 19} = 14.6$, $P < 0.01$	NS	NS
Day 14	$F_{1, 19} = 6.6$, $P < 0.05$	NS	$F_{1, 19} = 4.5$, $P < 0.05$

Supplementary Table S2. ANOVA results for change in body weight from day 1 in BALB/cJ mice. NS = not significant
