# Supplementary material for: Chronic Adolescent Restraint Stress Downregulates miRNA-200a Expression in Male and Female C57BL/6J and BALB/cJ Mice
Source: Genes (Basel). 2024 Jul 3;15(7):873. doi: 10.3390/genes15070873 (PMC11275362; doi:10.3390/genes15070873)
Supplement: Supplementary file 1 [file genes-15-00873-s001.zip › miRNA200a Table S3.pdf]

	Main Effect of Day	Main Effect of Sex	Day X Sex Interaction
Day 2	$F_{1, 20}$ = 18.3, P < 0.001	NS	NS
Day 3	$F_{1, 20}$ = 45.1, P < 0.001	NS	NS
Day 4	$F_{1, 20}$ = 37.3, P < 0.001	NS	NS
Day 5	$F_{1, 20}$ = 44.6, P < 0.001	$F_{1, 20}$ = 6.6, P < 0.05	NS
Day 6	$F_{1, 20}$ = 67.3, P < 0.001	$F_{1, 20}$ = 18.9, P < 0.001	NS
Day 7	$F_{1, 20}$ = 42.1, P < 0.001	$F_{1, 20}$ = 9.9, P < 0.01	NS
Day 8	$F_{1, 20}$ = 22.3, P < 0.001	$F_{1, 20}$ = 9.9, P < 0.01	NS
Day 9	$F_{1, 20}$ = 19.0, P < 0.001	$F_{1, 20}$ = 10.8, P < 0.01	NS
Day 10	$F_{1, 20}$ = 15.9, P < 0.001	$F_{1, 20}$ = 7.0, P < 0.05	NS
Day 11	$F_{1, 20}$ = 8.3, P < 0.01	$F_{1, 20}$ = 10.9, P < 0.01	NS
Day 12	$F_{1, 20}$ = 8.6, P < 0.01	$F_{1, 20}$ = 15.7, P < 0.001	$F_{1, 20}$ = 6.1, P < 0.05
Day 13	$F_{1, 20}$ = 10.3, P < 0.01	$F_{1, 20}$ = 10.7, P < 0.01	NS
Day 14	$F_{1, 20}$ = 10.1, P < 0.01	$F_{1, 20}$ = 9.3, P < 0.01	NS

Supplementary Table S3. ANOVA results for change in body weight from day 1 in C57BL/6J mice. NS = not significant
